# Transvaginal Evisceration in a Patient With Rectovaginal Prolapse: A Twist in the Tale

**DOI:** 10.7759/cureus.75134

**Published:** 2024-12-05

**Authors:** Sofia Leandro, Rita Leandro, Ana Martins, Artur Silva, Manuel Carvalho

**Affiliations:** 1 General Surgery, Hospital do Espírito Santo de Évora, Évora, PRT; 2 General Practice, Areeiro Family Health Unit, Lisbon, PRT

**Keywords:** emergency surgery, minor trauma, postmenopausal complications, rectovaginal prolapse, small bowel herniation, transvaginal evisceration, vaginal wall defect

## Abstract

Transvaginal evisceration is a rare, potentially life-threatening condition involving herniation of intra-abdominal contents, typically the small bowel, through a defect in the vaginal wall. Most commonly observed in postmenopausal women with a history of pelvic surgery or trauma, it necessitates prompt surgical intervention. We report a unique case of transvaginal evisceration in a 67-year-old postmenopausal female with rectovaginal prolapse following minor trauma. The patient presented with sudden pelvic pain and a mass protruding through the vaginal canal after lifting a heavy object. Examination revealed a loop of small bowel through the vaginal opening. She was hemodynamically stable, and initial management included bowel reduction and intravenous fluid administration. Emergency exploratory laparotomy confirmed a full-thickness defect in the posterior vaginal wall with approximately 40 cm of viable small bowel eviscerated. The defect was repaired with absorbable sutures, and the patient recovered uneventfully, being discharged on postoperative day five. This case underscores the importance of considering transvaginal evisceration in postmenopausal women with rectovaginal prolapse, even after minor trauma, to enable timely intervention and prevent severe complications.

## Introduction

Transvaginal evisceration is an uncommon but severe complication that involves the herniation of abdominal contents through a defect in the vaginal wall [1]. While this condition is often associated with prior pelvic surgeries, trauma, or vaginal cuff dehiscence, it can also occur spontaneously in postmenopausal women with atrophic vaginal walls. The occurrence of transvaginal evisceration in the context of rectovaginal prolapse is rare, with limited case reports in the literature [2]. Rectovaginal prolapse involves the descent of both the rectum and the vaginal wall, due to the weakened support structure that may predispose to evisceration, especially in the setting of increased intra-abdominal pressure. This report presents a unique case of transvaginal evisceration following minor trauma in a patient with a history of rectovaginal prolapse.

## Case presentation

**Figure 1:**
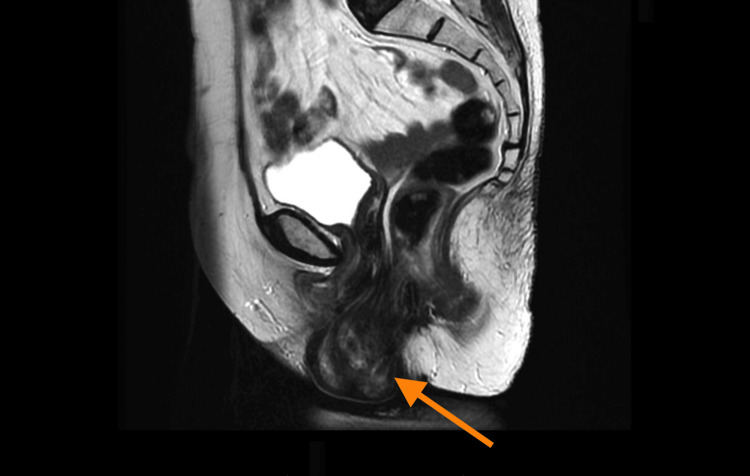
Preoperative MRI showing rectovaginal prolapse with herniation of abdominal contents into the pelvic cavity

A 67-year-old postmenopausal female with a known history of rectovaginal prolapse presented to the emergency department after experiencing a sudden onset of pelvic pain and the sensation of a mass protruding from her vaginal canal. The symptoms occurred after the patient lifted a heavy object [3]. Upon arrival, the patient reported severe discomfort and difficulty walking due to the prolapsed mass. She had no history of recent pelvic surgery, significant trauma, gastrointestinal complaints, or radiotherapy. The patient was under follow-up in a surgical consultation for her rectovaginal prolapse and had undergone a pelvic MRI two months prior to this episode (Figure 1). Additionally, she had a history of two vaginal deliveries.

**Figure 2:**
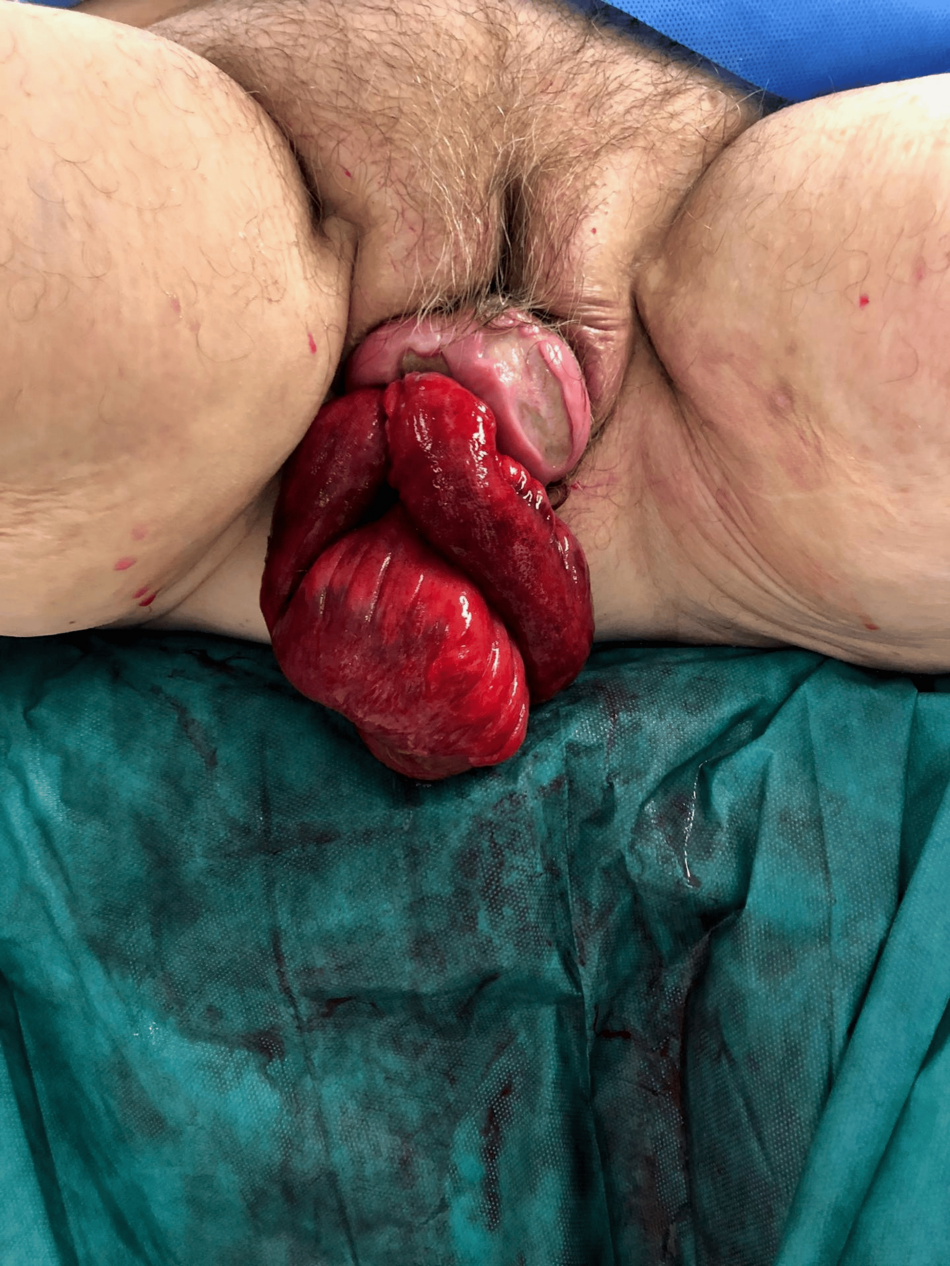
Transvaginal evisceration of small bowel in the patient with rectovaginal prolapse following minor trauma

On physical examination, a loop of small bowel was visualized protruding through the vaginal introitus (Figure 2). The eviscerated bowel appeared viable, with no signs of strangulation. The patient was hemodynamically stable, and initial management focused on preventing further injury to the bowel. Gentle manual reduction of the bowel was attempted with success, and intravenous fluids were administered to maintain hydration [2].

No CT scan was performed in the emergency setting. The patient was taken directly to the operating room for an urgent exploratory laparotomy based on the clinical findings.

**Figure 3:**
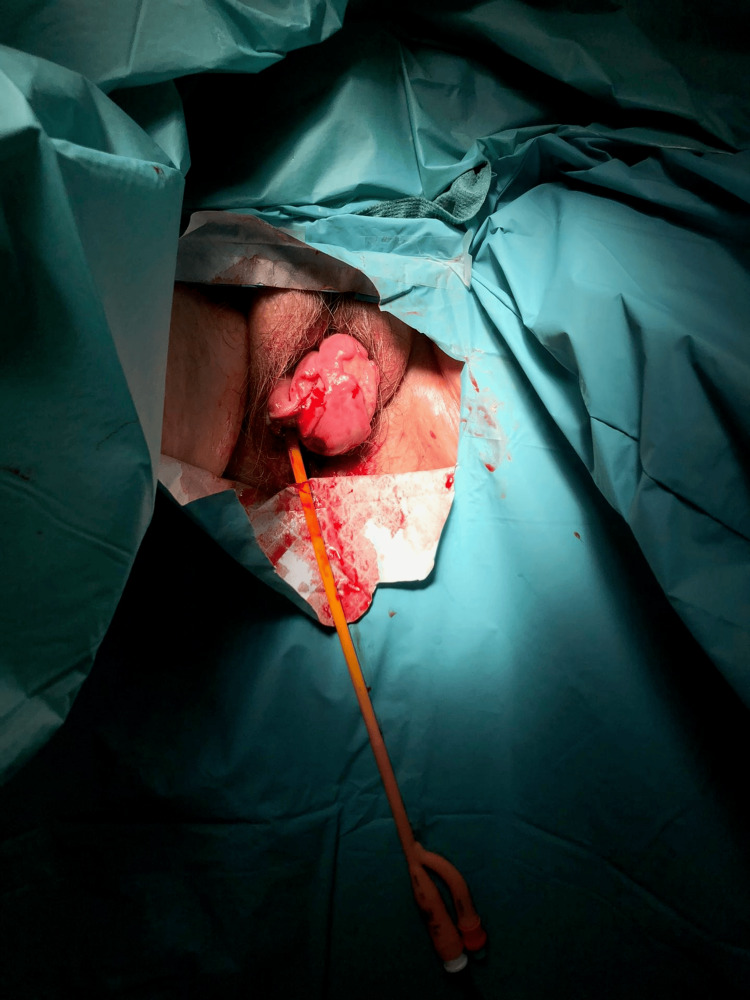
Intraoperative view after reduction of the eviscerated small bowel, with the vaginal wall defect prepared for repair

Intraoperatively, under general anesthesia, a full-thickness defect measuring approximately 4 cm was identified in the posterior vaginal wall. Approximately 40 cm of viable small bowel had eviscerated through this defect (Figure 3). The bowel was carefully reduced into the abdominal cavity, and the vaginal defect was repaired with absorbable sutures. The patient tolerated the procedure well, with no intraoperative complications.

Postoperatively, the patient was monitored closely for signs of bowel ischemia and infection. Her recovery was uneventful, and she was discharged on postoperative day five. At her six-week follow-up, the patient reported complete resolution of her symptoms and was referred to pelvic floor physical therapy for long-term management of her rectovaginal prolapse [4,5].

## Discussion

Transvaginal evisceration is a rare condition that requires prompt diagnosis and intervention to avoid life-threatening complications. The majority of cases occur in postmenopausal women with predisposing factors such as a history of pelvic surgery, pelvic radiation, vaginal atrophy, or trauma [6]. This case illustrates a unique scenario in which a Valsava maneuver triggered transvaginal evisceration in a patient with rectovaginal prolapse, a condition that already weakens the structural integrity of the pelvic floor (Figure 1).

The pathophysiology of transvaginal evisceration involves a combination of increased intra-abdominal pressure and a defect in the vaginal wall [1]. In postmenopausal women, vaginal atrophy due to estrogen deficiency weakens the vaginal wall, making it more susceptible to rupture. Additionally, the presence of rectovaginal prolapse further compromises the pelvic support system, increasing the likelihood of evisceration, even after the Valsava maneuver [3,4].

Management of transvaginal evisceration requires immediate surgical intervention to reduce the risk of bowel ischemia and sepsis [7]. In this particular case, an exploratory laparotomy was chosen over laparoscopy due to the urgency and complexity of the clinical presentation. The patient had a significant amount (approximately 40 cm) of small bowel eviscerated through a full-thickness defect in the vaginal wall. Given the visible protrusion of the bowel through the vaginal introitus and the potential risk of bowel ischemia, prompt open surgical intervention was deemed more appropriate to quickly assess the viability of the bowel and ensure a secure repair of the vaginal defect. Laparoscopy was not considered as it may have delayed the intervention and provided limited access for immediate reduction and repair of the extensive vaginal wall defect observed. In this case, early recognition and surgical repair of the vaginal defect led to a favorable outcome. The use of absorbable sutures for vaginal wall closure is recommended to reduce the risk of infection and promote healing. Postoperative care should include monitoring for signs of infection, bowel ischemia, and proper wound healing.

## Conclusions

This case highlights the importance of recognizing transvaginal evisceration as a potential complication in patients with rectovaginal prolapse, even in the absence of significant trauma. Prompt diagnosis and surgical intervention are essential to prevent severe complications. Clinicians should maintain a high index of suspicion when postmenopausal women with pelvic floor disorders present with acute pelvic pain and vaginal mass as early intervention is critical for successful outcomes.
